# Harvesting Ant—Cats as Letter Carriers—Antediluvian Rhinoceros—Flying Fish—Horse Notes—Birds and Musical Sounds—Breeding of Fish—Blood Stains—Texan Fever

**Published:** 1880-10

**Authors:** 


### MISCELLANEOUS.

VERY young fish are phosphorescent.—*Am. Naturalist.*

APROPOS of fertile hybrids, certain journals have stated that a mule in Paris has had six young—from a horse, ass, and a zebra.—*Am. Naturalist.* July, 1880.

THE HARVESTING ANT.—This is a small ant, the worker being about a line long, of a reddish-brown color. The males and females are several times longer than the workers. The female weighs about four times as much as the workers. Then there is, in the same community, the soldier ant, with an enormous head, furnishing brains for the whole formicary. In fighting powers they far surpass the cock sparrows; in their furious encounters they dismember each other, and yet persist in the contest, *sans* legs, *sans* eyes, *sans* bodies —*Am. Naturalist.*

SO ENGROSSING is the partiality of the domestic cat for its home, that certain Dutch naturalists have come to the sage conclusion that Grimalkin may be utilized as a letter carrier with considerable advantage to public interests. They propose to organize a service of post cats, and are at present engaged, by a series of ingenious experiments, in testing pussy's capacities for delivering the mails. Selecting Luik for their headquarters, they thence dispatched a number of cats, securely tied up in woollen bags, to the neighboring villages, where they are freed from confinement and turned loose, with neat packets of letters strapped firmly to their backs At once their domestic instincts come into full play, and they swiftly flee homeward with unswerving directness. Of thirty-seven cats, thus constrained to serve their country, not one has hitherto failed to fulfil its postal function with excellent punctuality. It is feared, however, that when a double service shall be arranged, difficulties and delays may arise from the meetings of post cats on the high road.

AN ANTEDILUVIAN RHINOCEROS.—German archæologists have been exceptionally lucky this summer in turning up old-world curiosities as well as relics of the prehistoric ages. Schleswig-Holstein only the other day afforded a copious yield of antique armor, huge and uncouth weapons of war, and mighty carved drinking-horns; and now we learn from Magdeburg that the eminent physiologist, Leo Baltzer, who has, for some time past, been carrying on excavations in the neighborhood of Nordhausen, has just made a highly interesting discovery in the alluvial stratum of the Steigerthal, close to the foot of Schellenberg. There, at the depth of about six feet below the surface of the valley, he came upon the entire skeleton of an antediluvian rhinoceros, in an excellent state of preservation. The height of this enormous beast, when living, must have exceeded that of the tallest guardsman in our Household Brigade, and his bulk can scarcely have been less than that of the average African elephant. The fore and hind legs, spinal column, ribs, and collar-bone are perfect. Some deterioration has accrued to the skull through the fact that it was several feet nearer the surface of the earth than were the extremities, and consequently suffered from the infiltration of rain-water. However, the jaws and twenty-eight of the teeth are quite uninjured. Dr. Baltzer is at present busily engaged in setting up the huge skeleton, which he intends, as soon as his task shall be completed, to present to the High School of Magdeburg.—*London Telegraph*, Sept. 11th, 1880.

---

DO FLYING FISH FLY?—C. C. Whitman answers in the negative, in the same number of the above-named journal. There are no muscles which operate upon their fins as those which perform the office of flying in birds

---

PROF. A. N. PRENTISS discourages the attempt to destroy obnoxious insects by means of fungoid growth, such as are grown by the use of yeast on plants. Mr. J. W. Burns, of Shelter Island, N. Y., succeeded in thus destroying potato beetles.—*American Naturalist*, September, 1880.

---

IN Prof Newton's article on the goose, in the "Encyclopædia," he remarks the predominance of the white variety in domestication is probably due in part to the practice of plucking the birds alive, for it is well known to bird-keepers that a white feather is often produced in place of one of natural color that has been plucked out. The reviewer adds that saddle galls on horses become covered with white hairs, and that he possesses a black cat which has a white star on its head where it was picked by a fowl in its kittenhood.—*American Naturalist.*

---

HORSE NOTES.—Mr. Robert Bonner, in a recent interview with a reporter, made the scientific statement that fast trotters must be as near the thorough-bred as is consistent with trotting action. Mr. Bonner has done more than any living man toward the development of the trotting-horse, and it has been done on scientific principles. It has been left, however, for Mr. Wm. H. Vanderbilt to reduce the marvellous time of the trotter to 2:11¾. Both of these gentlemen were shrewd enough to look to blood. If it could be ascertained positively, there is little doubt that every horse that has trotted below 2:30 was, and is from three-quarters to seven-eighths thoroughbred.

---

IT is quite impossible to doubt that birds are fond of musical sounds The song of nightingales and linnets, the deep notes of the South American bell-bird, the incessant cooing of the dove, the noisy chattering of the parrots, the ringing cry of the whippoorwill, all lead to the same conclusion. Here, again, these sounds are of precisely the same nature as those employed by the crickets, katydids, cicadas, and other musical insects, as well as by man himself in his vocal and instrumental music. Something of the same taste is displayed among the quadrumana by the howlers and other monkeys But it is a noteworthy fact that a large majority of these presumably sexual calls, in birds, insects, and other animals, are true musical sounds, not mere noises. I have pointed out elsewhere the probable reason for this preference of pure tones in the case of mankind; and the same argument will apply, *mutatis mutandis*, to all other animals. But there is certainly a singular analogy in this respect between sounds and colors, most animals preferring the relatively pure and simple musical tones to confused noises; and the relatively pure and simple analytic colors, red, blue, green, and yellow, to confused mixtures, such as brown, gray, and mud-color. At any rate, a bird evidently pays far more attention to the musical class of auditory perceptions than to mere noise A canary will take no notice of ordinary confused sounds in a room; but, if one begins to chirp or whistle to it, it immediately responds with another chirp in emulation. So, too, when a piano or other musical instrument is played in the neighborhod of a singing bird, it will often show its recognition of the musical character by pouring out its very fullest flood of song, as if to conquer its unconscious rival.—GRANT ALLEN, in *Popular Science Monthly*.

---

### THE BREEDING OF FISH.

### WHAT THE STATE COMMISSION WILL DO IN THE ABSENCE OF FUNDS.

*To the Public*:—The Governor of this State has declined to give his sanction to the usual appropriation for the maintenance of artificial fish culture, the means whereby the public waters of the State have been stocked with the choicest game and food fish to an extent that has created plenty where before there was scarcity This unlooked for action, the reason for which has not been communicated to the Commissioners of Fisheries, leaves a valuable public property in waters, buildings, hatching appliances, and stock fish, without the means of available use, or even of preservation. The stock fish, many thousands in number requiring daily care and feeding, and capable of producing annually 5,000 000 fry, are the fruit of many years' careful selection and breeding, and if lost now, cannot be replaced without years of labor and many thousands of dollars' expenditure The State hatchery at Caledonia, acquired at the cost of $15,000, is, in respect to its supply in quantity and quality of water, its equipment in the most approved appliances for breeding, its skilled and experienced operators, its supply of stock fish, and in the success which has attended all its operations the most complete and valuable of its kind on the west side of the Atlantic Ocean. What has been done by the commission toward replenishing exhausted waters with the finest game and food fish is well known to the public. The multiplication of shad in the Hudson River to the extent of many millions each year, so that the cost to consumers has been largely reduced within the last 10 years, alone justifies all the appropriations that have been made by the State for the promotion of artificial fish culture.

The commissioners cannot consistently with the duty committed to them by the Legislature, permit this valuable property and the important interest involved in its use, to go to decay and destruction by reason of the refusal of the Executive to sanction the apropriations necessary for its support. They therefore announce to the public that the operations of the State hatchery will, if need be and as far as shall be necessary to save the State from loss, be continued under the personal responsibility of the members of the commission, after the close of the fiscal year, and until the Legislature shall have an opportunity to review the action of the Governor, in the hope that in the enlightened sense of members of the law-making power will sustain them by making the necessary appropriations at the earliest possible stage of the session: or, failing to do this, will provide by law for such a disposition of the property as will save the sacrifice which may result from a failure to provide the requisite current support.

ROBERT B. ROOSEVELT, President.

RICHARD U. SHERMAN.

EUGENE G. BLACKFORD,

Commissioners of Fisheries.

Aug. 14, 1880.

BLOOD STAINS eighteen months old, were distinctly recognized by Prof. George Mees, M.D., and decided to be human blood by microscopic and other tests, and after the substance in which they were found had been repeatedly wet with perspiration. —*American Naturalist.*

PROF. LAW, of Cornell University, by direction of Governor Cornell, has made an examination of the cattle distemper in Oswego, N. Y., and declares it to be Texan fever.

